# Age-Dependent Changes in Protist and Fungal Microbiota in a Peruvian Cattle Genetic Nucleus

**DOI:** 10.3390/life14081010

**Published:** 2024-08-14

**Authors:** Richard Estrada, Yolanda Romero, Carlos Quilcate, Deisy Dipaz, Carol S. Alejos-Asencio, Silvia Leon, Wuesley Yusmein Alvarez-García, Diorman Rojas, Wigoberto Alvarado, Jorge L. Maicelo, Carlos I. Arbizu

**Affiliations:** 1 Dirección de Desarrollo Tecnológico Agrario, Instituto Nacional de Innovación Agraria (INIA), Lima 15024, Peru; richard.estrada.bioinfo@gmail.com (R.E.); yolanda.bioinfo@gmail.com (Y.R.); ceqp2374@yahoo.com (C.Q.); dipazdeysi8@gmail.com (D.D.); alejoscarito1@gmail.com (C.S.A.-A.); sleont@gmail.com (S.L.); walvarez@inia.gob.pe (W.Y.A.-G.); diormanr@gmail.com (D.R.); 2 Facultad de Ingeniería Zootecnista, Agronegocios y Biotecnología, Universidad Nacional Toribio Rodríguez de Mendoza de Amazonas (UNTRM), Cl. Higos Urco 342, Chachapoyas 01001, Peru; wigoberto.alvarado@untrm.edu.pe (W.A.);; 3 Facultad de Ingeniería y Ciencias Agrarias, Universidad Nacional Toribio Rodríguez de Mendoza de Amazonas (UNTRM), Cl. Higos Urco 342, Chachapoyas 01001, Peru

**Keywords:** microbial diversity, cattle, hematological parameters, gut microbiota

## Abstract

In this research, the connection between age and microbial diversity in cattle was explored, revealing significant changes in both protist diversity and fungal microbiota composition with age. Using fecal samples from 21 Simmental cattle, microbial communities were analyzed through 18S rRNA gene sequencing. Results indicated significant differences in alpha protist diversity among the three age groups, while fungal composition varied notably with age and was linked to hematological parameters. Despite the stability of fungal alpha diversity, compositional changes suggest the gut as a stable niche for microbial colonization influenced by diet, clinical parameters, and microbial interactions. All cattle were maintained on a consistent diet, tailored to meet the specific nutritional needs of each age group. These findings emphasize the importance of understanding age-related microbial dynamics to enhance livestock management and animal health, contributing to broader ecological and biomedical research. This study was limited by the lack of comprehensive metabolic analyses correlating microbiota changes with specific age-related variations, indicating a need for further research in this area.

## 1. Introduction

The gut microbiota constitutes a complex and diverse ecosystem, composed of various microorganisms such as bacteria, methanogenic archaea, ciliates, anaerobic fungi, viruses, and bacteriophages [1]. These symbiotic communities degrade dietary components like plant fibers, carbohydrates, proteins, and lipids, generating volatile fatty acids (VFAs) that are crucial for the animal, meeting up to 70–80% of its energy requirements [2]. The gut microbiome, closely related to various physiological functions of the host, is fundamental to the health and performance of livestock [3,4]. This complex system, influenced by factors such as diet, genetics, and the age of the host and its environment, has significant implications for the animal’s health and development [5,6].

The composition of the microbiota in ruminants varies significantly with age, influencing their health and metabolic processes [7]. In young cattle, the microbiota is diverse and becomes more complex as the animals mature, which is crucial for efficient digestion and overall health [8]. Specifically, in cattle, the ruminal microbiota changes with age, affecting the diversity and stability of intestinal microbes, impacting digestion and general health [9]. Diet continues to be the primary factor determining the structure and composition of the ruminal microbiota [10]. Clinical parameters such as glucose and cholesterol are linked to age-induced variations in the microbiota, reflecting metabolic adjustments as the animal matures [11]. Additionally, the diet of animals is influenced by geographical location and the type of production system. Scientific findings demonstrate that lipid supplements can reduce enteric methane production, providing viable options to mitigate the environmental impact of animal production [12].

The intestinal microbiota of cattle includes diverse fungal and protist communities [13,14]. Dominant fungal phyla in cattle gut microbiota are Ascomycota, Basidiomycota, and Neocallimastigomycota [15]. These fungal phyla play crucial roles in various gut functions [13]. Additionally, prominent protist phyla include Ciliophora and Apicomplexa, which are integral to nutrient metabolism and overall gut health [14,16].

The gut microbiota in cattle undergoes age-related changes, influencing various aspects of health, including metabolic, immune, and cognitive functions [17]. Probiotics play a significant role in modulating this microbiota, aiding in healthy aging by enhancing the intestinal barrier and improving nutrient absorption, particularly in older cattle [17]. Early probiotic supplementation aids juvenile animals, including newborn calves, by fostering development, improving gastrointestinal health, and boosting immunity to diseases [18]. Analyzing the microbiota from a genetic core perspective offers valuable insights into its inherited effects on well-being. This approach enables the creation of customized probiotic treatments tailored to various animal populations, enhancing their overall health and productivity [19]. The diet of cattle is also shaped by geographical location and production systems, which in turn affects the microbiota’s composition and function. Simmental cattle are especially recognized for their dual-purpose use in meat and dairy production, due to their fast growth and effective feed conversion. Contemporary genomic research has discovered genetic variants linked to meat quality, concentrating on genes associated with muscle development and growth rate [20,21]. Furthermore, Simmental cows are known for their strong milk production capacity, making them highly suitable for dual-purpose farming operations [22,23].

The 18S rRNA molecular marker is crucial for deciphering the intestinal microbiota of fungi and protists due to its conserved nature among eukaryotes, allowing precise identification and classification [24]. This marker, coupled with high-throughput sequencing technologies like Illumina, enables detailed profiling of microbial communities, including low-abundance species [25]. This comprehensive approach is essential for linking microbial diversity with host health, facilitating targeted probiotic therapies to enhance animal health and productivity [26].

This study investigates how age affects the intestinal microbiota of cattle, focusing on fungi and protists, and examines correlations with blood parameters across three age groups within a genetic core of healthy bovines. The findings reveal that age significantly affects the composition and diversity of the microbiota and its relationship with various clinical parameters. These results underscore the importance of comprehending the age-related dynamics of the intestinal microbiota and its impact on the metabolic health of cattle. Additionally, this study underscores the significance of identifying age-specific biomarkers and exploring personalized probiotic therapies to enhance cattle health and performance across various stages of life.

## 2. Materials and Methods

### 2.1. Animal and Sample Collection

In Huaral, Lima, at an altitude of 128 m above sea level (11°31′18″ S and 77°14′06″ W), 21 fecal samples were gathered from Simmental cattle at the Donoso Agricultural Research Station (EEA Donoso for its acronym in Spanish). This station hosts a government-owned herd that serves as a cattle genetic nucleus. The conditions included natural light exposure, a relative humidity of 88.5%, and an average temperature of 25.5 °C [27]. An initial pilot study was performed to ascertain the ideal number of replications needed for this research. The designated age groups were defined as 58 to 63 months for 5Y, 18 to 21 months for 1Y8M, and 5 months for 5M. The sex ratio across all age groups was 4 females to 3 males. The diet regimen at the Donoso Experimental Station of INIA (Peru) relies on fresh forage, with particular supplements tailored to the age group. Based on the dietary details provided in a previous study [28], the diet and composition details are provided in Supplementary Table S1. Stool samples were collected directly from the rectum of each animal using disposable gloves, transported to the laboratory in liquid nitrogen, and kept at −80 °C until DNA extraction. Additionally, blood samples were drawn from each animal’s jugular vein. Twenty-one cattle from EEA Donoso were included in this study. These animals, regularly monitored by the veterinary unit, underwent parasitological examinations revealing no cysts, oocysts, or larvae and were certified as healthy. Regular veterinary checks, such as physical examinations, clinical history assessments, and laboratory analyses, are carried out to maintain the stringent health standards necessary for semen and ovum donors. Consequently, no unhealthy animals exist in the genetic nucleus. This study adhered to Peruvian National Law No. 30407: “Animal Protection and Welfare”.

### 2.2. Clinical Parameters

According to the methodology implemented in a prior investigation [28], the clinical parameter abbreviations are detailed in Supplementary Table S2.

### 2.3. DNA Extraction and 18S rRNA Gene Sequencing

Total genomic DNA was extracted from the 21 fecal samples using a commercial kit (Norgen, Biotek Corporation, Sacramento, CA, USA) following the manufacturer’s recommended protocol. To evaluate the quality of the extracted DNA, its concentration was measured with the NanoDrop ND-1000 spectrophotometer (Nanodrop Technologies, Wilmington, DE, USA), while the 260/280 absorbance ratio was also determined. Furthermore, DNA integrity was verified through 1% agarose gel electrophoresis, ensuring the reliability of the extracted samples. To construct the Illumina amplicon sequencing library, around 10 ng of DNA from each sample was used for PCR amplification with the 528F/706R primer pair targeting the 18S rRNA gene. The PCR procedure began with an initial denaturation at 94 °C for 2 min. This was followed by 5 cycles of denaturation at 94 °C for 45 s, annealing at 52/54 °C for 45 s, and extension at 72 °C for 1 min. An additional 35 cycles were then carried out, with a reduced annealing temperature of 50/52 °C. The final step was an elongation at 72 °C for 10 min. The library was prepared using the Illumina TruSeq DNA PCR-Free Library Preparation Kit (Illumina, USA), in accordance with the manufacturer’s guidelines, incorporating index sequences. Library quality was subsequently assessed using a Qubit 2.0 Fluorometer (Thermo Scientific, Waltham, MA, USA). The approved libraries were eventually subjected to sequencing on the 250-bp paired-end Illumina NovaSeq 6000 platform (Illumina Inc., San Diego, CA, USA) following the manufacturer’s guidelines.

### 2.4. Bioinformatics Analysis

In the QIIME2 [29] analysis, trimming and quality filtering were performed. Following this, the paired-end reads, demultiplexed by Illumina, were analyzed with the Qiime2-DADA2 [30] software v2023.9, leading to the creation of an Amplicon Sequence Variant (ASV) table. To minimize the occurrence of erroneous ASVs, any sequence exhibiting fewer than 10 reads across all samples was discarded. Plant sequences were specifically removed. Taxonomic classification of ASVs was performed using the SILVA v138.1 database for identifying fungi and protists through 18S sequence analysis. The high-quality sequences, after filtering, were then aligned using the integrated MAFFT [31] aligner. Using the QIIME2 phylogenetic module, rooted and unrooted phylogenetic trees for fungi and protists were constructed with the FastTree algorithm.

### 2.5. Statistical Analysis

The data underwent statistical evaluation using the packages Phyloseq (v1.223) [32], MicrobiotaProcess, and Microeco [33] in R software v4.1.1 [34]. Individual samples underwent analysis to generate rarefaction curves, assessing the sequencing depth. Subsequently, various measures of intestinal bacterial alpha diversity were evaluated (Pielou, Simpson, ACE, Observed, Chao1, and Shannon indices). Beta diversity was assessed using the Jaccard and Unweighted UniFrac methods, with the results visualized through Principal Coordinate Analysis (PCoA). To evaluate differences in microorganism communities among groups, a two-way PERMANOVA [35] test was conducted, utilizing 9999 permutations. Distinctive features in gut microbiota profiles were detected using the LEfSe method, which applies linear discriminant analysis (LDA) effect size. This method highlighted biomarkers with the highest statistical and biological significance. Spearman rank correlation tests were conducted on pairs of variables, including clinical parameters and fungal alpha diversity indices. Additionally, the relationship between clinical variables and fungal community composition was examined using Mantel tests with 999 permutations.

## 3. Results

For fungi, a total of 1,808,136 high-quality reads were generated, with an average of 86,101 high-quality reads, a maximum of 171,671 high-quality reads, and a minimum of 20,340 high-quality reads. Similarly, for protists, a total of 1,189,559 high-quality reads were obtained, with an average of 56,645 high-quality reads, a maximum of 144,851 high-quality reads, and a minimum of 4288 high-quality reads.

### 3.1. Impact of Age on the Diversity and Composition of Fungal and Protist Communities in the Bovine Microbiome

**Figure 1:**
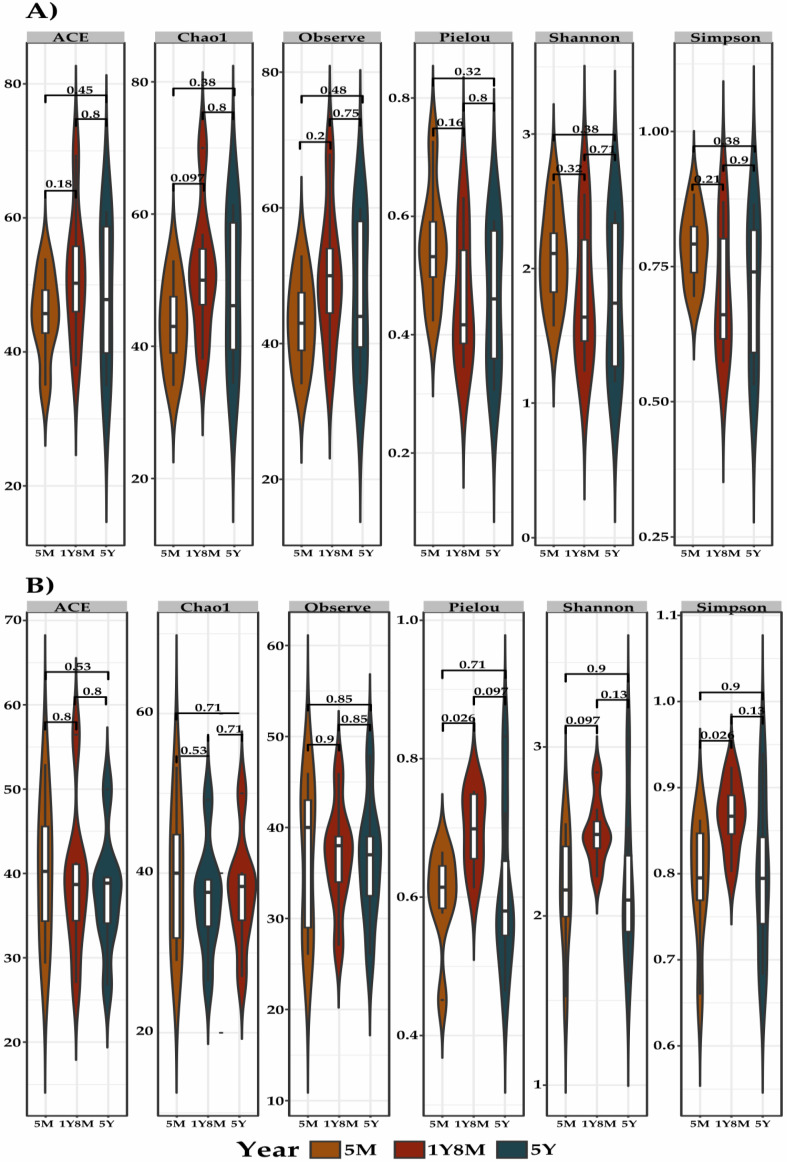
Alpha diversity (Observed, Pielou, Shannon, and Simpson) gut microbiota indices between cattle in three age groups: 1Y8M, 5Y, and 5M. (**A**) Gut microbiota index of fungal alpha diversity. (**B**) Protist alpha diversity gut microbiota index. The values indicated are *p*-values.

The rarefaction curve demonstrated good representativity of species diversity in the analyzed samples, indicating that the sampling was adequate for the analysis of both fungi (Figure S1) and protists (Figure S2). The alpha diversity of fungal and protist communities in cattle exhibited distinct patterns (Figure 1). For fungal communities, measured through the same metrics, no significant differences were observed (Figure 1A). In contrast, the alpha diversity of protist communities, measured through the metrics ACE, Chao1, Observed, Pielou, Shannon, and Simpson, exhibited significant differences between age groups. In particular, Pielou (*p* = 0.026) and Simpson (*p* = 0.026) metrics presented significant differences between the age groups 1Y8M and 5M (Figure 1B).

**Figure 2:**
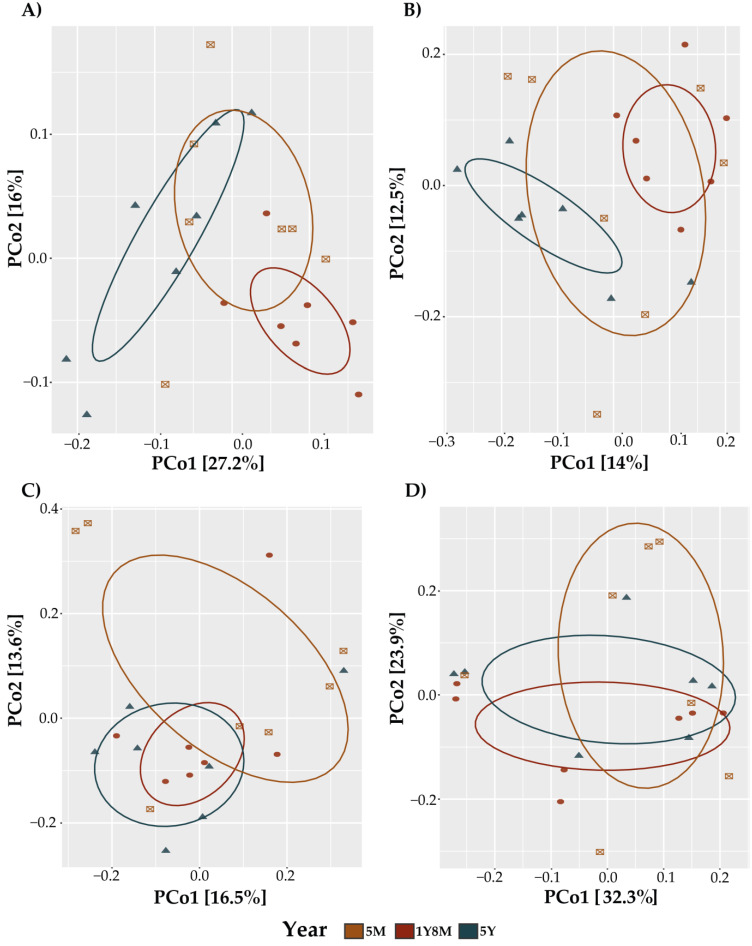
Principal Coordinate Analysis (PCoA) plot of beta diversity based on Jaccard (**A**,**C**) and Bray–Curtis (**B**,**D**) distance. (**A**) PCoA using Jaccard distance of fungi. (**B**) PCoA using Bray–Curtis distance of fungi. (**C**) PCoA using Jaccard distance of protists. (**D**) PCoA using Bray–Curtis distance of protists.

In the analysis of the beta diversity of the fungal communities in cattle, significant patterns influenced by age and the interaction of age and sex were observed. The PCoA plots for both metrics indicated distinct clustering of fungal communities across different age groups (Figure 2A,B). The PERMANOVA analysis for Jaccard distances (Table 1) indicated that age (*p* = 0.015) and the interaction of age and sex (*p* = 0.007) significantly influenced the beta diversity. Similarly, the PERMANOVA for Unweighted UniFrac distances (Table 1) demonstrated significant effects of age (*p* = 0.002) and the interaction of age and sex (*p* = 0.002). For the protist community, using both the Jaccard index and Unweighted UniFrac, PCoAs were generated to display the similar distribution of the samples across different age groups (Figure 2C,D). The permutational multivariate analysis of variance (PERMANOVA) revealed no significant disparities between the evaluated groups (Table 1).

**Figure 3:**
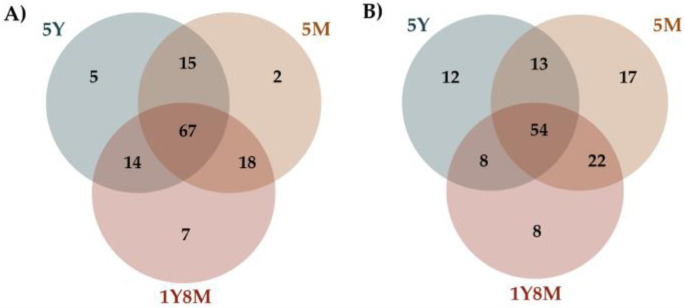
Venn diagrams presenting the shared and unique taxa among different age groups of cattle. (**A**) Fungal Venn diagram. (**B**) Protist Venn diagram.

Venn diagrams illustrated the distinct and overlapping fungal and protist ASVs among cattle of different age ranges (Figure 3). For fungal ASVs (Figure 3A), 67 are common across all age groups, with 7 unique to the 1Y8M group, 5 to the 5Y group, and 2 to the 5M group. Shared ASVs between two age groups include 14 between 1Y8M and 5Y, 18 between 1Y8M and 5M, and 15 between 5Y and 5M. For protist ASVs (Figure 3B), 54 are common across all age groups, with 8 unique to the 1Y8M group, 12 to the 5Y group, and 17 to the 5M group. Shared ASVs include 8 between 1Y8M and 5Y, 22 between 1Y8M and 5M, and 13 between 5Y and 5M.

### 3.2. Taxonomic Composition of the Fungal and Protist Communities in the Gut Microbiota

**Figure 4:**
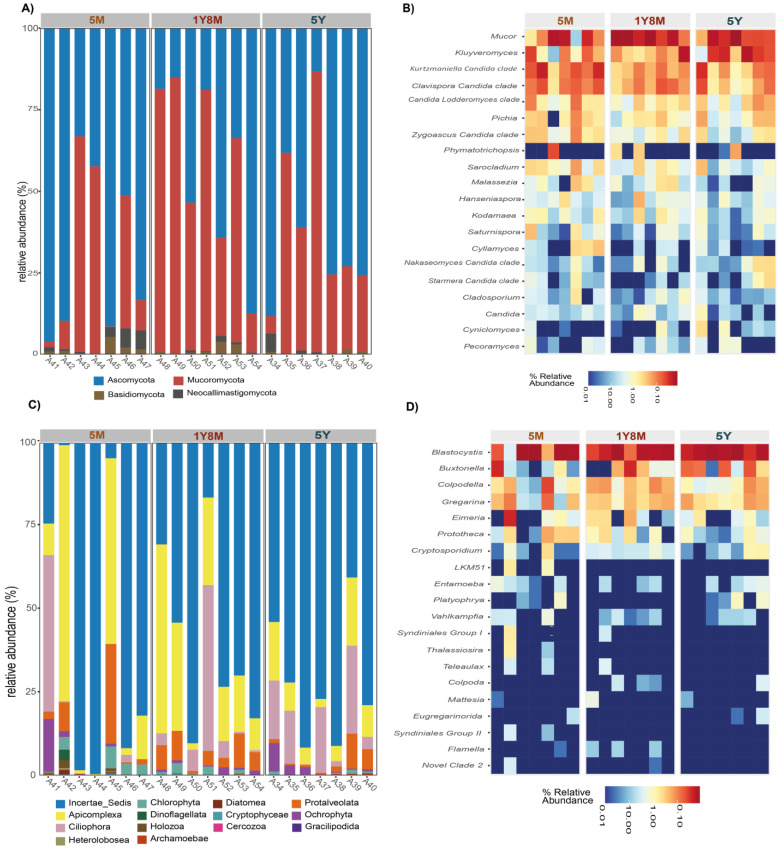
Comparative analysis of fungal and protist communities across different age groups of cattle. (**A**) Bar plots representing the relative abundance of fungal taxa. (**B**) Heatmaps presenting the distribution and abundance of the 20 most abundant fungal genera. (**C**) Bar plots representing the relative abundance of protist taxa. (**D**) Heatmaps presenting the distribution and abundance of the 20 most abundant protist genera.

The analysis of the intestinal fungal microbiological structure in cattle revealed a predominance of the phyla Ascomycota and Mucoromycota across different ages (Figure 4A). Ascomycota had a relative abundance of 75%, 80%, and 80% in cattle aged 1Y8M, 5Y, and 5M, respectively. Mucoromycota exhibited an abundance of 20%, 15%, and 25% at the same ages. Basidiomycota had lower representation with values of 3%, 3%, and 2%, while Neocallimastigomycota presented low percentages of 2%, 2%, and 3%.

Similarly, the intestinal microbiological structure of protists exhibited significant variations across different ages (Figure 4C). Incertae_Sedis was the predominant phylum at all ages, with relative abundances of 75%, 80%, and 75% in cattle aged 1Y8M, 5Y, and 5M, respectively. Apicomplexa demonstrated relative abundances of 20%, 15%, and 25%, while Ciliophora displayed 15%, 10%, and 10%. Other phyla, such as Protalveolata, Chlorophyta, and Ochrophyta, maintained lower percentages, generally below 5%. These findings indicate a clear predominance of Incertae_Sedis and a notable presence of Apicomplexa and Ciliophora, with other phyla exhibiting relatively low but consistent abundances across different ages.

The heatmap displays the proportional representation of diverse protist and fungal genera across various samples, highlighting both the most and least abundant genera across different age groups (Figure 4B,D). In the fungi (Figure 4B), *Mucor*, *Kluyveromyces*, *Kurtzmanella-Candida* clade, *Clavispora-Candida* clade, *Pichia*, and *Candida-Lodderomyces* clade were the most abundant genera in the intestinal microbiota of cattle at different ages, exhibiting high relative abundances. In contrast, genera such as *Phymatotrichopsis*, *Sarocladium*, *Malassezia*, *Hanseniaspora*, *Kodamaea*, *Saturnispora*, *Cylamyces*, *Nakaseomyces-Candida* clade, *Starmera-Candida* clade, *Cladosporium*, *Candida*, *Cyniclomyces*, and *Pecoramyces* were the least abundant, with low relative abundances in all age groups. With respect to protists (Figure 4D), *Blastocystis*, *Buxtonella*, *Colpodella*, and *Gregarina* were the most abundant genera, displaying high relative abundances across all age groups. In contrast, genera such as *LKM51*, *Entamoeba*, *Platyophrya*, *Vahlkampfia*, *Syndiniales* Group I, *Thalassiosira*, *Teleaulax*, *Colpoda*, *Mastigina*, *Eugregarinorida*, *Syndiniales* Group II, *Flamella*, and Novel Clade 2 were the least abundant, with low relative abundances at all ages.

### 3.3. Biomarkers Identified Based on Age in Fungi and Protists

**Figure 5:**
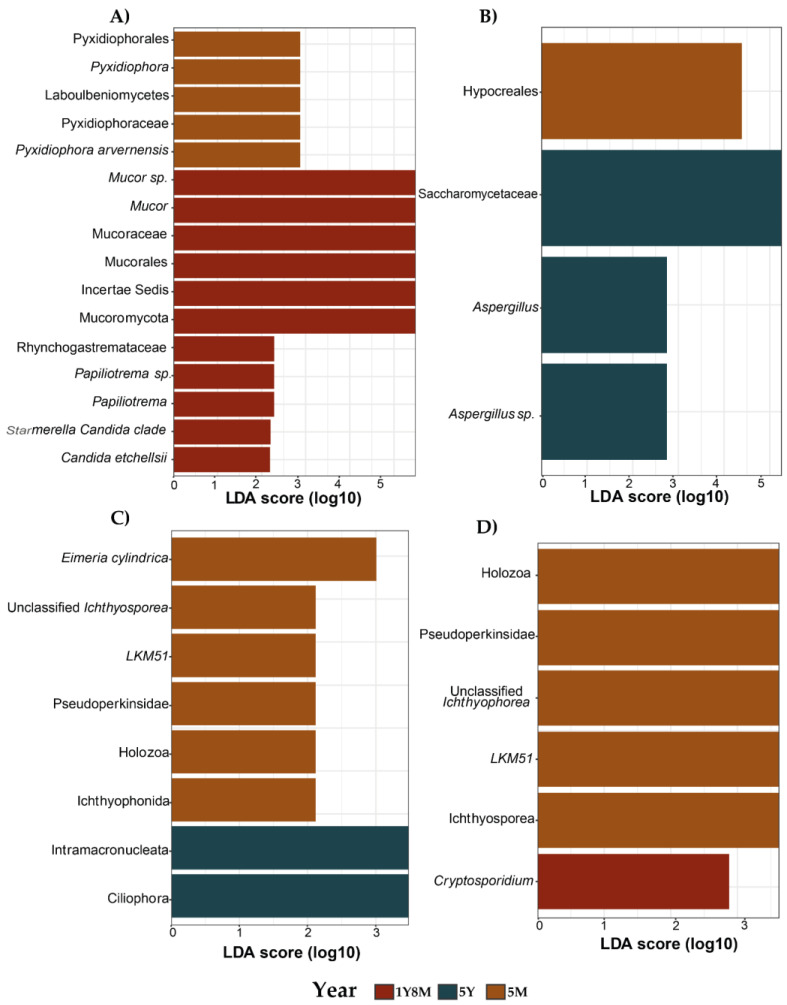
Differences in the intestinal fungal (**A**,**B**) and protist (**C**,**D**) microbiota among different ages in cattle. Bar chart illustrating the differentially abundant taxa identified through linear discriminant analysis (LDA). (**A**) Differentially abundant fungal taxa in the 1Y8M and 5M group. (**B**) Differentially abundant fungal taxa in the 5Y and 5M group. (**C**) Differentially abundant protist taxa in the 5M and 5Y group. (**D**) Differentially abundant protist taxa in the 5M and 1Y8M group.

To identify specific fungal and protist taxa associated with different age groups, a comparative analysis of fecal microbiota compositions was performed utilizing the LEfSe method approach (Figure 5). The taxa exhibiting the most significant differences, from phylum to genus level, were identified based on their LDA scores. For fungi, the 1Y8M group demonstrated enrichment in two phyla (Incertae Sedis and Mucoromycota), one order (Mucorales), two families (Mucoraceae and Rhynchogastremataceae), two genera (*Mucor* and *Papiliotrema*), and four species (*Mucor* sp., *Papiliotrema* sp., *Candida etchellsii*, and *Starmerella Candida* clade). In the 5Y group, the analysis indicated enrichment in one order (Pyxidiophorales), one class (Laboulbeniomycetes), one family (Pyxidiophoraceae), one genus (*Pyxidiophora*), and one species (*Pyxidiophora arvernensis*) (Figure 5A). For the 5M group, the analysis demonstrated enrichment in one order (Hypocreales). In contrast, the 1Y8M group exhibited enrichment in one family (Saccharomycetaceae), one genus (*Aspergillus*), and one species (*Aspergillus* sp.) (Figure 5B).

For protists, the 5Y group exhibited enrichment in two phyla (Ciliophora and Intramacronucleata). In contrast, the 5M group demonstrated enrichment in one family (Pseudoperkinsea), one order (Ichthyophonida), one class (Holozoa), and three genera (*Eimeria cylindrica*, unclassified *Ichthyosporea*, and *LKM51*) (Figure 5C). Additionally, for the 5M group, there was further enrichment in two classes (Holozoa and Ichthyosporea), one family (Pseudoperkinsea), and two genera (unclassified *Ichthyophorea* and *LKM51*). The 1Y8M group exhibited enrichment in one genus (*Cryptosporidium*) (Figure 5D).

### 3.4. Correlation of Alpha and Beta Diversity of Fungi with Clinical Parameters

**Figure 6:**
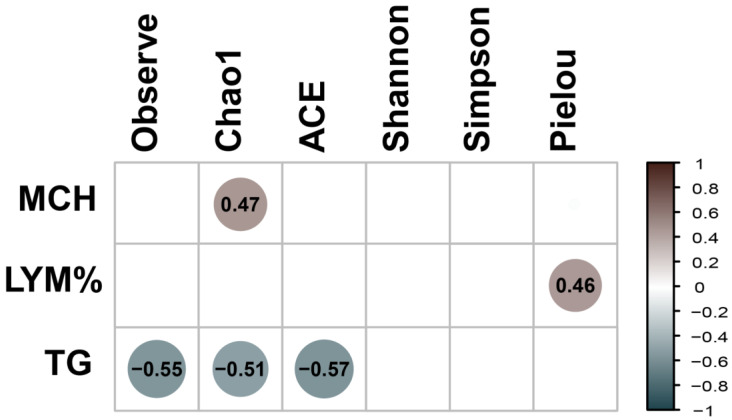
Clinical parameters and fungal alpha diversity were analyzed using Spearman correlations. Positive correlations are indicated in brown, while negative correlations are represented in dark blue. The matrix includes only statistically significant correlations (*p*-values < 0.05).

Analyses of clinical parameters (Table S3) were performed using the Kruskal–Wallis test, considering age as the variable of interest. The parameters that were significant for age were RBC, MCV, MCHC, MCH, LYM%, NEU, SEG, LYM, EOS, and TP (Table S4). Subsequently, Bonferroni post hoc analysis was conducted. In the 5Y-5M comparison, significant differences were observed in RBC, MCV, MCHC, MCH, NEU, SEG, LYM, EOS, LYM%, and TP. Similarly, in the 1Y8M-5M comparison, significant differences were detected in RBC and TP. Finally, in the 1Y8M-5Y comparison, significant differences were identified in MCV and MCH (Table S5). A Spearman correlation analysis was conducted to examine the relationship between clinical parameters and fungal alpha diversity indices (Figure 6). The Observed, ACE, and Chao1 indices were significantly negatively correlated with TG. The Pielou index was significantly positively correlated with LYM%. The Chao1 index was significantly positively correlated with MCHC.

Several significant results were identified in the correlation analysis of clinical variables with beta diversity for fungi (Table 2). The Mantel test using the Jaccard index revealed significant correlations for MCV (*p* = 0.03) and MCH (*p* = 0.02). The Partial Mantel test also demonstrated significant correlations for MCV (*p* = 0.014) and MCH (*p* = 0.01). Additionally, the Mantel test with the Unweighted UniFrac index indicated significant correlations for MCH (*p* = 0.045) and LYM% (*p* = 0.048). The Partial Mantel test did not reveal any significant correlations.

## 4. Discussion

In this study, the relationship between age and microbial diversity in cattle was investigated, revealing that protist diversity and the composition of the fungal microbiota change significantly with age. Variations were observed in the fungal composition and its relationship with hematological parameters. These findings are crucial as they highlight the dynamic nature of the gut microbiota and its interaction with host physiology over time.

Age did not exhibit a significant correlation with fungal alpha diversity, which is consistent with previous findings in tigers [36] and goats [37]. Conversely, age was significantly correlated with fungal composition, a phenomenon also reported in other mammals, such as humans [38,39] and monkeys [40]. This could be due to the intestine providing a relatively stable niche for fungal colonization [41], where predominant species establish early and maintain their presence throughout the host’s life [42]. On the other hand, fungal composition may be more influenced by changes in the host’s internal environment as it ages, such as dietary alterations, immune changes, and interactions with other microbiotas [43]. These factors can affect which fungal species are more abundant or dominant at different life stages, without necessarily changing the total number of species present [44].

In the present study, notable variations in the Simpson and Pielou indices of alpha diversity of protists were observed across the 1Y8M cohort and the 5M cohort, but not when comparing the 5Y group and the 5M group. This observation suggests the importance of considering age as a complex and non-linear factor in microbiota diversity. The first two years of life represent a critical developmental period during which the gut microbiota undergoes significant changes, which could explain the observed difference between 1Y8M and 5M [45]. After this period, alpha diversity tends to decrease, where in the early stages of life there is a rapid growth in alpha diversity, but the growth rate gradually decreases as age increases, as demonstrated in studies of microorganisms in cattle [46] and other mammals such as humans [47,48]. Furthermore, factors such as environment and individual physiology can influence these results, suggesting that the relationship between age and alpha diversity is more complex [49]. Conversely, no significant differences were observed in protist beta diversity. Although specific studies on the relationship between age and alpha protist diversity are scarce, some research has reported significant differences in protist microbiota diversity in age-related contexts. For instance, lower eukaryotic diversity has been documented in patients with Parkinson’s disease [50], and it is suggested that eukaryotic biomass and diversity may be influenced by lifestyle and diet in populations of different ages [51]. Furthermore, a study in alpacas identified significant differences in the diversity of alpha protists between different age groups and health states [52]. These findings underscore the need for studies specifically focused on the influence of age on eukaryotic diversity to better understand how this factor can affect the microbiota in different species and contexts.

The fungal phyla prevalent across the three age categories are comparable, encompassing Ascomycota, Mucoromycota, Basidiomycota, and Neocallimastigomycota. These results align with previous research on cattle and other ruminant species, including bovines [16,53], goats [54], and alpacas [55]. Similarly, the analysis of protists revealed that the dominant phyla were Incertae Sedis, Apicomplexa, and Ciliophora, which have also been reported in humans [51,55] and primates [56]. In this study, Incertae Sedis includes *Blastocystis* sp., which is predominant among the identified genera. This finding has also been reported in cattle [57], goats [58], camels [59], and humans [60].

The most abundant genus identified and recognized as a biomarker in the 1Y8M cattle group was *Mucor*. This aligns with studies indicating that this genus is more prevalent in non-obese individuals and its abundance increases with weight loss, suggesting a favorable metabolic health state [61]. This genus was also common in the human gastrointestinal tract, associated with intestinal health [62], and has been reported in a higher proportion in adults than in young people [63]. Furthermore, in yaks with diarrhea, *Mucor* was not detected, indicating that its growth is restricted in the presence of diarrhea [64]. The genus *Blastocystis* was the most prevalent genus in this study and is known to confer beneficial effects on the host immune system, such as stimulating mucus production through the cytokine IL-22, which improves intestinal health and alleviates colitis symptoms [60]. Additionally, recent studies have associated *Blastocystis* colonization with greater bacterial diversity in the gut microbiota, indicative of a healthy microbiota [60,65]. The presence of *Blastocystis* correlates negatively with Bacteroides levels and positively with increased bacterial diversity, which is often linked to better health and reduced risk of inflammatory conditions [66]. These findings suggest that the high prevalence of *Mucor* and *Blastocystis* in the three age groups of healthy cattle could be related to a balanced and beneficial gut microbiome.

In the 5Y age group, *Aspergillus* was identified as a biomarker. This genus is common in the gastrointestinal tract of several animals and has been associated with beneficial roles such as fiber digestion. It also has potential pathogenic implications under certain conditions [67]. Metabolites produced by *Aspergillus* may play a role in maintaining a beneficial microbial balance in the absence of disease-triggering factors [68]. The presence of *Aspergillus* in healthy cattle may be related to its ability to interact beneficially with other microbial species, promoting a balanced and healthy intestinal microbiome [68].

In the 1Y8M group, *Eimeria cylindrica* was identified as a biomarker. Despite its presence, the animals did not exhibit clinical symptoms, suggesting that under specific conditions, this pathogen can be present without causing disease [69]. This observation aligns with other studies that have detected *Eimeria* spp. in cattle without clinical symptoms, indicating that factors such as host immunity and environmental conditions can influence the pathogenicity of these parasites [70,71]. In the 5M age group, *Cryptosporidium* was identified as a distinctive biomarker. This genus *Cryptosporidium* is generally associated with severe diarrhea in young ruminants [72]. Its presence in asymptomatic animals has been reported in lambs with *Cryptosporidium xiaoi* and *Cryptosporidium ubiquitum* [73]. This highlights the variability in the pathogenicity of *Cryptosporidium* depending on the species and the host’s immune status [74,75]. The detection of *Cryptosporidium* in asymptomatic individuals underscores the need to interpret its presence in diagnostic and epidemiological studies with caution, as it does not always indicate active disease [76,77].

A significant negative correlation was identified between alpha diversity indices Observe, Chao1, and Ace and triglycerides, suggesting that lower fungal diversity is associated with higher triglyceride levels. Certain fungi can help maintain healthy triglyceride levels [61]. The abundance of *Mucor racemosus* has also been observed to significantly influence fasting triglyceride levels, suggesting its potential as a biomarker for cardiovascular risk [78]. Furthermore, hypertriglyceridemia in older individuals has been associated with a reduction in gut mycobiome diversity [79]. A significant positive correlation between fungal alpha diversity and lymphocytes suggests that higher gut fungal diversity is associated with increased lymphocyte counts, highlighting the importance of gut mycobiome homeostasis for host immune functions [80]. For instance, colonization by fungi such as *Candida albicans* can stimulate the proliferation of Th17 cells and IL-17 feedback, aiding in the fight against infections [81]. Additionally, a reduction in intestinal fungi has been correlated with decreases in immune factors in the blood, such as lymphocytes, underscoring the protective role of symbiotic fungi in calibrating the immune response [82]. A significant positive correlation has also been observed between fungal alpha diversity Chao1 and MCHC, which is positively associated with DNA levels and intestinal colonization by *Candida albicans* [83]. Since MCHC reflects the concentration of hemoglobin in red blood cells, these results suggest that greater fungal diversity could be linked to better hematological function.

Correlation analysis demonstrated that several hematological parameters, such as MCV and MCH, are significantly correlated with fungal beta diversity. These correlations suggest that variations in the composition and hemoglobin content of red blood cells could be linked to alterations in the intestinal mycobiota composition. Previous studies have indicated that hematological health can influence the gut mycobiota, affecting both the diversity and abundance of certain fungal species [84]. Furthermore, fungal dysbiosis has been linked to inflammatory conditions and hematological diseases, highlighting the interaction between hematological status and intestinal health [85]. This underscores the importance of investigating how variations in hematological parameters influence the diversity and functionality of the intestinal mycobiota, which could have therapeutic implications for improving intestinal homeostasis and systemic health [38,85].

This study does not provide a comprehensive metabolic analysis linking microbiota changes to specific age-related variations in fungi and protists. Further studies are required to explore the metabolic pathways and their interactions with microbiota components at various life stages. This underscores crucial areas for future investigation to deepen our understanding of the complex relationships among microbiota, aging, and reproductive status.

## 5. Conclusions

This study examined how age influences microbial diversity in cattle and found that the composition and diversity of the gut microbiota change with age. While the alpha diversity of fungi remained constant, that of protists showed significant variations between age groups only in the Simpson and Pielou indices, suggesting the need for further research to clarify the impact of protists on the intestinal microbiota. Significant changes were observed in the composition of fungal communities but not in protists, and fungi composition was associated with some hematological parameters. Spearman correlations indicated a negative relationship between triglycerides and certain mycobiota diversity indices and a positive relationship between lymphocytes and fungal alpha diversity. These results suggest a dynamic interaction between fungal microbiota and host physiology over time. Additional studies are needed to correlate microbiota changes with age-related variations through comprehensive metabolic analyses. These findings underscore the importance of understanding how microbial dynamics evolve with age to enhance cattle management and health, given the uniform diet composition with varying concentrations across all cattle.

## Figures and Tables

**Table 1:** PERMANOVA of Jaccard and Unweighted UniFrac. * *p* < 0.05, ** *p* < 0.01

Items	Df	SumOfSqs	R^2^	F	Pr (>F)
Jaccard					
Year	2	0.397308	0.130516	1.472821	0.015 *
Sex	1	0.197202	0.064781	1.462057	0.055
Year/Sex	2	0.426412	0.140076	1.580706	0.007 **
Residual	15	2.023202	0.664625		
Total	20	3.044126	1		
Unweighted UniFrac					
Year	2	0.16684	0.20852	2.7976	0.002 **
Sex	1	0.04198	0.05247	1.4079	0.168
Year/Sex	2	0.14402	0.18	2.4149	0.002 **
Residual	15	0.44728	0.55901		
Total	20	0.80012	1		

**Table 2:** Correlation of clinical parameters with beta diversity (Jaccard and Unweighted UniFrac) using Mantel and Partial Mantel Tests. Only significant variables are presented. * *p* < 0.05.

	Jaccard				Unweighted UniFrac			
	Mantel Test		Partial Mantel Test		Mantel Test		Partial Mantel Test	
Variables	r	*p*	r	*p*	r	*p*	r	*p*
MCV	0.196815555	0.03 *	0.209922522	0.014 *	0.137675537	0.07	0.104071883	0.124
MCH	0.177721795	0.022 *	0.192394859	0.01 *	0.149206478	0.045 *	0.114890751	0.082
LYM%	0.192477483	0.064	0.194205056	0.068	0.195319744	0.048 *	0.178444189	0.094

## Data Availability

The raw data supporting the conclusions of this article will be made available by the authors on request.

## Supplementary Materials

The following supporting information can be downloaded at: https://www.mdpi.com/article/10.3390/life14081010/s1, Figure S1: Rarefaction curves of species richness illustrate the sequencing depth of fungi data obtained from gut samples. Figure S2: Rarefaction curves of species richness illustrate the sequencing depth of protist data obtained from gut samples. Table S1: Diet and composition. Table S2: List of abbreviations. Table S3: Clinical parameters of three age groups. Table S4: Kruskal–Wallis results for clinical parameters in cattle by age. Table S5: Post hoc Bonferroni results for clinical parameters in cattle by age.
