# Supplementary material for: Age-Dependent Changes in Protist and Fungal Microbiota in a Peruvian Cattle Genetic Nucleus
Source: Life (Basel). 2024 Aug 14;14(8):1010. doi: 10.3390/life14081010 (PMC11355802; doi:10.3390/life14081010)
Supplement: Supplementary file 1 [file life-14-01010-s001.zip › Supplementary figures.pdf]

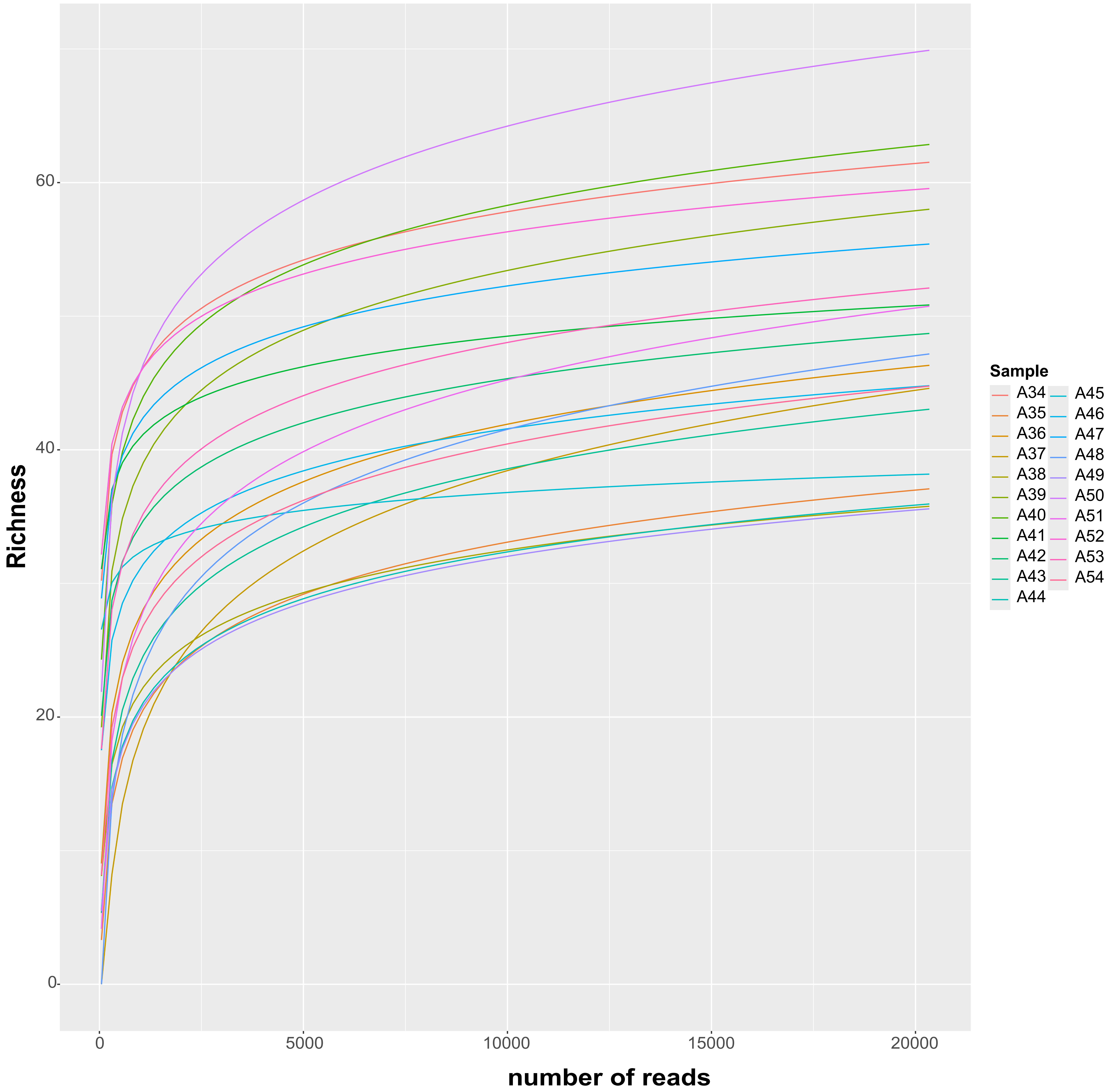

Figure S1: Species richness rarefaction curves show sequencing depth of 18S data obtained from fungi from gut samples

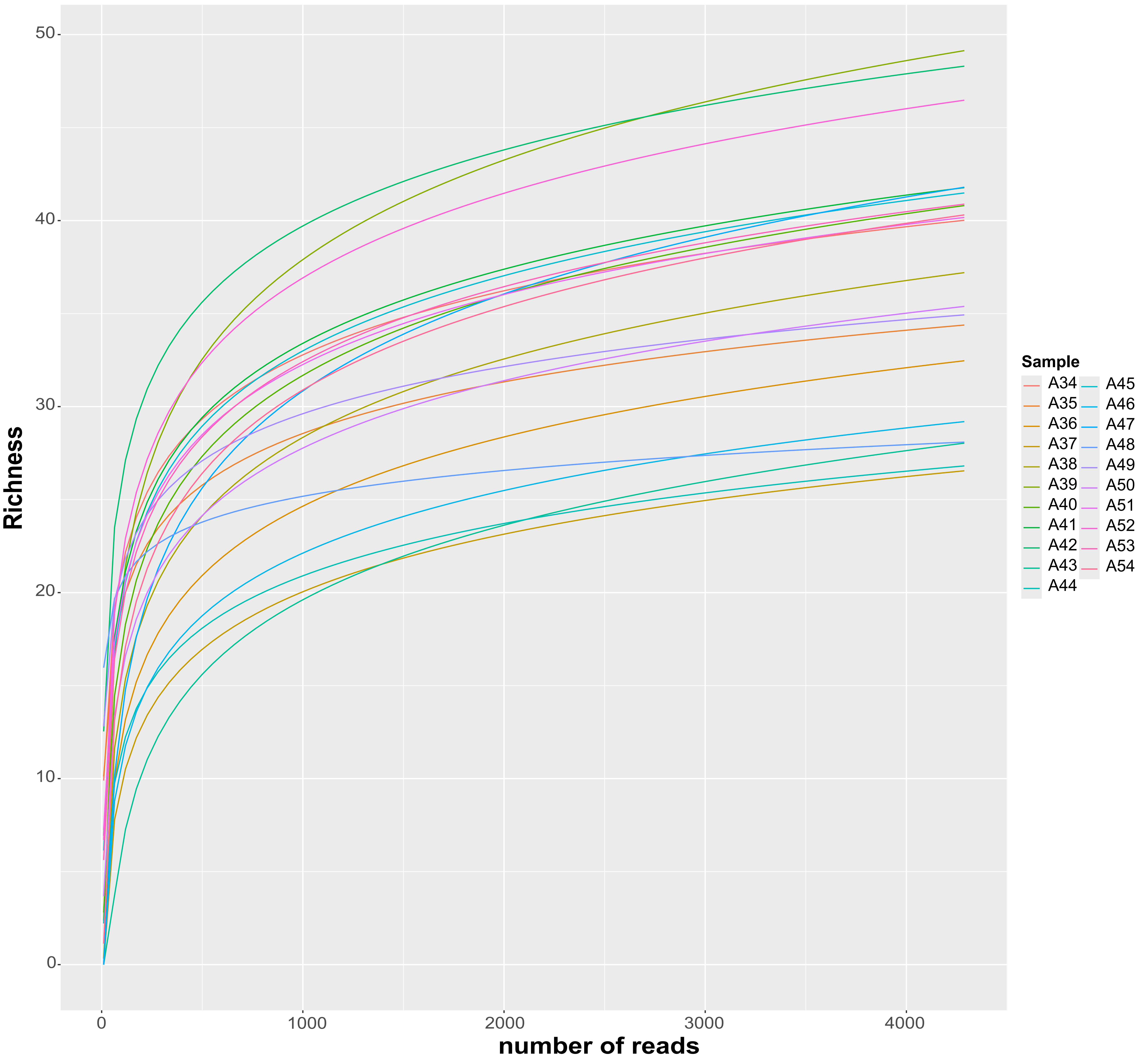

Figure S2: Species richness rarefaction curves show sequencing depth of 18S data obtained from protist from gut samples
